# Efficient organisation of intensive care units with a focus on quality: the non-physician provider

**DOI:** 10.1186/s13054-017-1703-4

**Published:** 2017-07-27

**Authors:** Herman Gerhard Kreeftenberg, Sjaak Pouwels, Peter Henricus Johannes van der Voort

**Affiliations:** 1 Department of Intensive Care Medicine, Catharina Hospital, Michelangelolaan 2, PO box 1350, 5601 ZA Eindhoven, The Netherlands; 2 Department of Surgery, Fransicus Gasthuis & Vlietland, Vlietlandplein 2, 3118 JH Schiedam, The Netherlands; 3 Department of Intensive Care Medicine, Onze Lieve Vrouwe Gasthuis, Oosterpark 9, PO box 95500, 1090HM Amsterdam, The Netherlands; 4 TIAS school for business and Society, Tilburg University, Warandelaan 2, PO box 90153, 5000LE Tilburg, The Netherlands

**Keywords:** Non-physician provider, ICU management, Quality of care, Physician shortage, Europe

Adequate staffing in intensive care units (ICUs) is an increasing problem worldwide. We would like to elaborate on staffing problems that arise in ICUs across Europe, including the Dutch situation.

This staffing problem is caused by the increased use of ICUs because of expanding and aging populations and economic welfare [1]. Also, migration to cities leaves several rural areas in Europe deprived of physicians. This is superposed by an increased demand from society for high quality health care.

This problem affects the availability of intensivists, residents and nurses. In North America, solutions for the same problem are being explored. To alleviate physicians’ workloads and to compensate for physician shortage, acute non-physician providers (NPPs) were introduced. They are physician assistants (PAs) and nurse practitioners (NPs) working on the ICU and performing the full scope of work residents do. They are supervised by an intensivist. Non-inferiority studies show that these NPPs are able to provide this care without decreasing quality. A review of these studies by Kleinpell et al. [2] showed promising results regarding the use of NPPs on the ICU.

Some countries in Europe, such as the Netherlands, have virtually no shortage of physicians. Still, the pressure from society to improve the quality of care leads to the question of whether NPPs should be used. In addition, work-hour restrictions for residents and the short duration of internships on the ICU make continuity in ICU care a struggle. This continuity of care is directly associated with quality [3]. As a result, in the Netherlands acute NPPs have been implemented in several ICUs. These NPPs graduated from ICU nurse to NPP after an additional two-year course. They know the ICU from their nursing specialty background. In contrast to residents, the step from nurse to NPP is often their final career move and therefore the personnel turnover in this NPP group is usually limited. In addition, because of their thorough knowledge of processes in the ICU, they seem the ideal candidates to achieve a high adherence to local standards and protocols.

Remarkably, in contrast to North America, European literature on this subject is lacking. Because of the promising results in the American literature and in the preliminary use of NPPs in European ICUs, we believe that challenges in this area of research for Europe lie in performing well-designed studies and implementation models to further explore the value of NPPs.

## Abbreviations

ICU: Intensive care unit
NP: Nurse practitioner
NPP: Non-physician provider
PA: Physician assistant

## References

[1] Cooper RA, Getzen TE, Laud P (2003). Economic expansion is a major determinant of physician supply and utilization. Health Serv Res..

[2] Kleinpell RM, Ely EW, Grabenkort R (2008). Nurse practitioners and physician assistants in the Intensive care unit: an evidence-based review. Crit Care Med.

[3] Cook RI, Render M, Woods DD (2000). Gaps in the continuity of care and progress on patient safety. BMJ.

